# Honey: Another Alternative in the Fight against Antibiotic-Resistant Bacteria?

**DOI:** 10.3390/antibiotics9110774

**Published:** 2020-11-04

**Authors:** Patricia Combarros-Fuertes, José M. Fresno, Maria Manuela Estevinho, Mário Sousa-Pimenta, M. Eugenia Tornadijo, Leticia M. Estevinho

**Affiliations:** 1 Department of Food Hygiene and Technology, Faculty of Veterinary Science, University of León, Campus de Vegazana, 24071 León, Spain; pcomf@unileon.es (P.C.-F.); jmfreb@unileon.es (J.M.F.); metorr@unileon.es (M.E.T.); 2 Department of Biomedicine, Unit of Pharmacology and Therapeutics, Faculty of Medicine, University of Porto, 4200-319 Porto, Portugal; mmestevinho@med.up.pt; 3 Department of Onco-Hematology, Portuguese Institute of Oncology of Porto (IPO-Porto), 4200-072 Porto, Portugal; mario.sousa.pimenta@ipoporto.min-saude.pt; 4 Centro de Investigação de Montanha (CIMO), Instituto Politécnico de Bragança, 5300-252 Bragança, Portugal

**Keywords:** natural antibiotic, antimicrobial compounds, antibacterial action mechanisms, medical uses, honey-resistance, honey limitations

## Abstract

Antibacterial resistance has become a challenging situation worldwide. The increasing emergence of multidrug-resistant pathogens stresses the need for developing alternative or complementary antimicrobial strategies, which has led the scientific community to study substances, formulas or active ingredients used before the antibiotic era. Honey has been traditionally used not only as a food, but also with therapeutic purposes, especially for the topical treatment of chronic-infected wounds. The intrinsic characteristics and the complex composition of honey, in which different substances with antimicrobial properties are included, make it an antimicrobial agent with multiple and different target sites in the fight against bacteria. This, together with the difficulty to develop honey-resistance, indicates that it could become an effective alternative in the treatment of antibiotic-resistant bacteria, against which honey has already shown to be effective. Despite all of these assets, honey possesses some limitations, and has to fulfill a number of requirements in order to be used for medical purposes.

## 1. Introduction

In 1945, Alexander Fleming, Howard Florey, and Ernst Chain were awarded the Nobel prize for the discovery of the first broad-spectrum antibiotic in history: penicillin. Soon after this finding, they realized and warned of the ease with which bacteria could develop tolerance to that new remedy if it was misused [1]. Today, antimicrobial resistance has become a challenging situation, not only for human health, but also for human-connected animals, farms, food, water, and natural ecosystems worldwide [2,3,4,5,6].

Antibiotic resistance is a natural phenomenon that occurs when bacteria are exposed to antibiotics. Under the drug selective pressure, susceptible bacteria are killed or inhibited, while those bacteria that are naturally resistant, or that have acquired antibiotic-resistant features, have more opportunities to survive and multiply [3]. The overuse and improper use of antibiotics amplify and accelerate this circumstance.

Currently, there are bacteria which are able to resist almost all, or even all, the approved antimicrobial agents for their treatment. Consequently, some common infections have become very difficult, or even impossible, to treat [3,7,8]. The cost of treating antibiotic-resistant infections is much higher than that of treating susceptible ones [9]; the rise in the costs of the antibiotic therapy, derived from the necessity of using more expensive antibiotics, is coupled with a lengthier stay in hospital [3,5].

Further to the economic impact, an increase in mortality is another consequence of antibiotic resistance. Recent estimations based on data from EARS-Net (European Antimicrobial Resistance Surveillance Network) show that each year, approximately 33,000 people in Europe die as a direct consequence of infections caused by bacteria resistant to antibiotics [2].

Therefore, the research and development of a new generation of antimicrobials to alleviate the expansion of antibiotic resistance has become a priority [7]. However, testing a new antimicrobial before commercialization requires long time periods. Moreover, the current strategies to limit the appearance of resistance and to safeguard the efficacy of new antimicrobials entail a significant obstacle in drug development. Limiting their use, as much as possible, to those cases that do not respond successfully to existing products leads to a lack of profitability for pharmaceutical companies. In this way, the development of new antimicrobials is no longer an interesting activity [10].

Faced with this situation, the search for new alternatives has led to the scientific study of substances, formulas or active ingredients used before the antibiotic era. Honey has been traditionally used not only as a food but also with therapeutic purposes. One of the most common curative applications is the topical treatment of wounds, especially for its antimicrobial properties [11,12]. However, with the advent of antibiotics, the use of honey gradually declined. In recent decades, more and more scientific studies have been focused on revealing which compounds present in honey are related to its antibacterial activity, and the action mechanisms through which honey kills bacteria. The effectiveness of manuka honey as an antimicrobial agent has been extensively studied; for this reason, it has long been regarded as one of the most efficacious honey varieties known. However, several studies using other honey varieties also demonstrated promising antibacterial properties, and prove similar, or even greater, efficacy than manuka honey [13]. Despite the multiple studies on the antibacterial properties of honey, its use as an antibacterial agent continues to be underestimated.

The purpose of this review is to provide a comprehensive and updated overview of the scientific evidence that supports the use of honey as a potential antimicrobial alternative against drug-resistant bacteria, either alone or in combination with currently used chemotherapeutic agents. This work describes, briefly, the components responsible for the antibacterial effects of honey, the mechanisms of action that have been found to date, as well as the main studies that have demonstrated the efficacy of honey against resistant bacteria. In addition, it compiles the assays aimed at evaluating the possible appearance of honey-resistance, and the limitations which may prevent its use for medical applications.

## 2. Factors Involved in Antibacterial Activity of Honey

Honey is a natural sweet substance constituted by hundreds of compounds [13,14,15]. Honey composition is inherently rather variable and depends mainly on the floral source, although certain external factors, such as seasonal, environmental, as well as processing, manipulation, packaging and storage conditions, also play an important role [15,16].

Some inherent characteristics, as well as a variety of substances which are part of honey composition, have been suggested as key elements responsible for its antimicrobial potential.

### 2.1. Phisicochemical Characteristics with Antibacterial Effects

#### 2.1.1. Osmotic Pressure and Low Water Activity

Honey is an oversaturated solution of sugars including fructose, glucose, maltose, sucrose and other minor sugars, such as rhamnose, trehalose or erlose, which represent around the 80% (*w/v*) of its composition. Water is the second largest constituent of honey and may vary under normal conditions from 15% to 20% (*w/v*) depending on the botanical origin and the level of maturity of the honey, as well as on the processing and storage conditions [15,17].

The high concentration of sugars combined with the low moisture content are responsible for the low water activity values of honey, which range from 0.562 and 0.62 [17,18], and cause osmotic stress in microorganisms [15,19]. Osmotic pressure induces the loss of water inside the bacteria, and once dehydrated, cells are unable to grow and proliferate [17]. In addtion, it has been suggested that the sugars present in honey interfere in bacterial quorum sensing [20,21], and more recently, it has been also demonstrated that osmotic pressure affects the ability of bateria to form biofilms [22].

Although a high concentration of sugar and a low water activity hamper the growth of many microorganisms, several studies have verified that artificial honey, prepared using a mixture of mono-and disaccharides at the same concentrations as those found in honey, is not as effective as honey in inhibiting bacterial growth [12,13,23,24,25].

#### 2.1.2. Acidity and Low pH

More than thirty-two organic acids have been identified in honey, including acetic, butyric, citric, formic, fumaric, glyoxylic, propionic, lactic, maleic, malic, oxalic and succinic acids [26]. However, gluconic acid, produced by the action of glucose oxidase on glucose, is the predominant one [27]. Due to the presence of these organic acids, honey is an acidic food, whose low value of pH (between 3.2 and 4.5), creates an unfavorable environment for microbial growth [28,29].

### 2.2. Compounds with Antibacterial Effects

Honey contains several compounds in minor proportions which have been related to its bioactive properties. These substances can be specific to a variety of honey, or may be found in variable concentrations among different honey samples, depending primarily on their botanical origin [30].

#### 2.2.1. Hydrogen Peroxide

Hydrogen peroxide (H_2_O_2_) is produced when the enzyme glucose oxidase (added by honey-bees to the nectar), is activated on moderate dilution of honey, and reacts with glucose, producing gluconic acid and H_2_O_2_ [19,31]. The maximum H_2_O_2_ concentration is reached at different dilutions according to the varieties of honey, but in most of honey samples, it occurrs at concentrations ranging from 15% to 50%. In general, dark honeys often produce higher amounts of H_2_O_2_ than lighter ones, but the multifactorial process influencing the production and decomposition of H_2_O_2_ makes difficult to predict its concentration [32].

H_2_O_2_ is considered a true antibacterial, as it produces minimum inhibitory concentrations in bacteria in the range 10–1000 µg/mL [31,33]. The contribution of H_2_O_2_ to the antibacterial activity has been assesed in several studies, which demostrated that H_2_O_2_ removal using catalase significantly reduces the antibacterial potential of honey [33,34,35]. However, H_2_O_2_ levels in honey solutions are too low to have any direct antibacterial activity, so additive or synergistic mechanisms with other honey compounds are necessary to explain its activity [36]. The degradation of H_2_O_2_ catalyzed by ions such as Cu^+^ or Fe^2+^, naturally present in honey, produces hydroxyl radicals (via Fenton reaction), which are more responsible for DNA damage than endogenous H_2_O_2_ [35,37]. Moreover, H_2_O_2_ hydrolysis also produces oxygen, which promotes the auto-oxidation of polyphenols by acting, under these circumstances, as pro-oxidant molecules responsible for bacteriostatic and DNA-damaging activities. Finally, H_2_O_2_ can react with benzoic acid, one of the phenolic acids present in honey, producing peroxy-acids, which are more stable and powerful antimicrobial agents than H_2_O_2_, and are able to resist the catalase activity [35,37].

Although H_2_O_2_ is widely considered as the primary source of the antibacterial activity of honey, there are varieties whose main antibacterial mechanisms are non-H_2_O_2_ dependent.

#### 2.2.2. Phenolic Acids and Flavonoids

Phenolic compounds are one of the largest groups of secondary metabolites that plants mainly synthesize for protection against biotic and abiotic stress and oxidative damage. These compounds are transferred via the nectar to honey. The phenolic compounds present in honey mainly belong to two families: phenolic acids and flavonoids [15,27].

Recent studies revealed that polyphenols are key components in the antimicrobial effects of honey [38,39,40]. The qualitative and quantitative dissimilarities observed in the phenolic profile of different honey varieties could justify the variations in their bioactivity [30,41]. In addition, the botanical origin may affect the profile of polyphenolic compounds, sufficiently to permit the discrimination among honey samples based on the predominance of some individual components [30,42,43].

The antibacterial activity of many flavonoids found in honey has already been described, as well as their main antibacterial action mechanisms [44,45,46], which in some cases are similar to those described for honey [14,47]. Nevertheless, their concentration in honey are not enough to produce, individually, the effects observed, and therefore their antibacterial properties are not due to their isolated action, but to their synergistic effects with other polyphenols, or other compounds, such as H_2_O_2_ [34,37]. Further investigations aimed at discovering the role of polyphenolic compounds and their direct antimicrobial impact are required.

#### 2.2.3. Methylglyoxal

As was previously mentioned, there are honey varieties whose antibacterial activity is unrelated to the H_2_O_2_ content. Non-peroxide activity has been attributed to a variety of different compounds, among which are those known as 1,2-dicarbonyls. The 1,2-dicarbonyls are strongly reactive substances originating from caramelization or Maillard reactions in carbohydrate-rich foods [14].

Methylglyoxal (MGO) is a 1,2-dicarbonyls-breakdown-product of antimicrobial significance, and it has been identified as the main antibacterial compound in manuka honey [14]. The high levels of MGO found in this variety of honey are formed by the spontaneous dehydration of dihydroxyacetone, which is present at unusually high concentrations in the nectar of *Leptospermum* genus, including *Leptospermum scoparium*, *Leptospermum polygalifolium*, and some related *Leptospermum* species native to New Zealand and Australia [48,49,50]. Originally, it was assumed that manuka honey was the only variety that presents MGO in its composition. Nevertheless, several studies have evidenced that MGO is a compound that is also present in other types of honey, such as mire and polyfloral Nordic honey [51], different monofloral honey varieties (citrus, eucalyptus, acacia, chestnut, lime, rhododendron, strawberry tree, sulla, sunflower and thyme), honeydew and polyfloral honey from Italy [52], Finnish polyfloral honey samples [53], and honeydew Portuguese honey [54]. However, the concentrations of MGO in these honey varieties ranged between 0.2 and 166 mg/kg, while in manuka honey, this compound is found, in general, in significantly higher concentrations (ranging from 38 to 1541 mg/kg) [19].

The antibacterial action mechanisms of MGO have not been completely elucidated, but go through inducing alterations in the structure of bacterial fimbriae and flagella, which would limit bacteria adherence and motility, as well as producing damage to cell membranes, and the shrinking and rounding of cells [55].

#### 2.2.4. Bee Defensin-1 Peptide

Bee defensin-1 is an antimicrobial peptide (AMP) found in bee hemolymph and is produced in their salivary glands, so it is incorporated into honey during the primary processing. Similar to other AMPs, such as apidaecin, abaecin or hymenoptaecin, defensin makes up part of bee the immune system [56,57,58]. The presence of bee defensin-1 in honey has not yet been systematically investigated, and widespread quantitative data of this peptide have not yet been established. It has been detected in the medical-grade honey Revamil, and in manuka honey it appears to be modified, which is thought to be the result of interaction with MGO, although its concentration in this variety is not significant [59,60,61]. Defensin-1 has been also detected in different varieties of honey from several regions of Slovakia, and more recently in eucalyptus honey samples from Ecuador [22,62]. The content of defensin-1 was variable among the honey varieties, and curiously in samples with similar floral sources, but different geographical origins, which indicates that defensin-1 content might be associated with the genetic diversity of honeybees [62].

Defensin-1 displays activity against different microorganisms, including Gram-positive and Gram-negative bacteria; however, its efficacy against multi-drug resistant bacteria seems to be limited at the concentration present in honey [59,60]. The antibacterial action mechanisms of bee defensin-1 have not been completely elucidated, but it is suggested that they might act by creating pores within the bacterial cell membrane, resulting in cell death, as happens with other species’ defensins [14]. Moreover, it has recently demonstrated that defensin-1 plays an important role in the antibiofilm activity of honey [22].

## 3. How Honey Acts against Bacteria: Antibacterial Action Mechanisms

**Figure 1:**
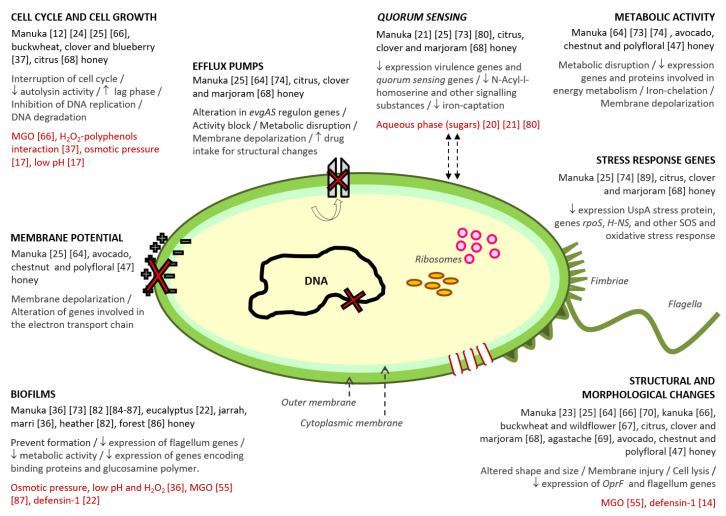
Antibacterial action mechanisms described for honey. Within each mechanism, the following information has been collected: (i) varieties of honey in which the mechanism was detected; (ii) the different effects related to the mechanism; (iii) the compound/s or honey characteristics associated to the mechanism, and (iv) the reference number of the studies from which the information was extracted.

Despite the numerous studies on the antibacterial properties of honey, the lack of comprehensive evidence explaining the mechanisms through which honey interferes with bacteria, limits, partly, its application as an antibacterial agent [12,63,64]. Honey is a very complex substance, containing hundreds of compounds that cause specific and distinct effects on microorganisms (Figure 1). The mode of action of honey against bacteria differs among Gram-positive and Gram-negative microorganisms, some studies hypothesized that at least some cellular targets might be broadly specific for each class of bacteria [12,13,23]. However, given the complexity of the matrix, there are probably several mechanisms that have not been described yet.

In terms of understanding the mechanisms of action of honey upon bacteria, most of the research has been undertaken using manuka honey. Nevertheless, more and more studies are being carried out with other, different varieties (Figure 1).

### 3.1. Structural and Morphological Changes

One of the earlier attempts to reveal honey’s action mechanisms were centred on observable characteristics by using scanning and transmission electron microscopy. In methicillin-sensitive *Staphylococcus aureus* and methicillin-resistant *S. aureus* (MRSA), it was verified that manuka honey did not induce a significant cellular lysis, few surface changes were found and the majority of the cells retained a smooth surface after four hours of treatment [12]. On the contrary, *Pseudomonas aeruginosa* cells exposed to manuka honey exhibited widespread structural damage and large membrane bubbles, which led to cell lysis and bacterial death [23]. This result was verified by genomic analysis, showing that manuka honey treatment causes a reduction in the expression of *OprF*, an integral membrane protein that is essential for the structural stability of the cell envelope in Gram-negative bacteria [65]. After these studies, it was found that manuka and kanuna honey exerted very different changes on the cellular morphology of *Bacillus subtilis*, *Escherichia coli*, *S. aureus,* and *P. aeruginosa.* While for the first three bacteria a significant cell shortening was observed, in the case of *P. aeruginosa,* cells appeared longer than usual, which is possibly a reflection of the cell envelope damage that likely happened [66]. More recently, flow cytometry also confirmed the previous results. The treatment time and the honey concentration were identified as key factors in the induction of membrane injury in *S. aureus* and *E. coli* cells. The differences observed among Gram-positive and Gram-negative bacteria were also highlighted in this study [64]. Membrane permeabilization after manuka honey exposition was also verified by flow cytometry assays in *P. aeruginosa* [25].

These mechanisms are not exclusively attributable to manuka honey, and several honey varieties have been demonstrated to produce morphological and structural alterations on bacteria as one of their first effects (Figure 1). Buckwheat honey and wildflower honey were demonstrated to induce a rapid disruption of the cell wall of *E. coli,* which was honey concentration- and treatment time-dependent [67]. Structural and morphological changes, such as altered shape, modified surface layers, cellular debris, increased vacuoles, and/or with irregular shrunken cytoplasm, as well as the presence of electron dense material, were also described in *E. coli* cells after treatment with citrus, clover and marjoram honey [68]. Agastache honey caused extensive cell lysis and membrane disruption in MRSA and *E. coli* [69]. Avocado, chestnut and polyfloral honey samples induced membrane injury upon *S. aureus* and *E. coli* with differences among bacterial types. While the effect of the different honey samples on *S. aureus* membrane integrity was limited, in the case of *E. coli* cells, the results obtained confirm a more meaningful damage. Under similar treatment conditions, these three honey samples caused more relevant changes in membrane integrity than manuka honey [47]. On the other hand, no morphological changes in *P. aeruginosa* cells were observed after Corsican honey exposure, despite the fact that this bacterium was sensitive to the different varieties tested [35].

In addition, related to changes in bacterial morphology, it was verified that manuka honey suppresses *P. aeruginosa* flagellum gene expression, by impacting on regulatory and structural genes, which entails a reduction in protein expression, and means a significant reduction in flagellated cells. This finding has consequences beyond morphological alterations in bacterial cells, since flagellum is critical for pathogens to establish and produce invasive infection, so honey can also reduce the pathogenic potential of infecting bacteria [70].

### 3.2. Alterations of Bacterial Membrane Potential

Membrane potential plays an essential role in several bacterial physiological processes. The loss of the equilibrium of ion concentration inside and outside of bacteria may affect to their viability [71].

Three recent studies described, for the first time, that different honey varieties (manuka, avocado, chestnut and a polyfloral honey) induce significant membrane depolarization in *P. aeruginosa*, *S. aureus*, and *E. coli* [25,47,64]. This finding has great significance, since a collapse in the membrane potential means that bacteria are not able to generate the energy required to carry out their normal physiological processes, such as the correct spatial organization of cell division proteins and regular cell division function [72], as well as to drive the mechanisms necessary for its survival, such as some multidrug efflux pumps [25] (Figure 1).

### 3.3. Bacterial Cell Cycle and Cell Growth Changes

The first studies using scanning and transmission electron microscopy revealed that the main effect of manuka honey upon methicillin-sensitive *S. aureus* and MRSA involved the interruption of the cell cycle. Bacterial cells were unable to separate, which resulted in an accumulation of arrested cells with a fully formed septum [12]. It was suggested that septa were formed prematurely in the cell cycle, and cell division was then interrupted because mandatory cellular events had not been completed yet. Alternatively, cell division might have been prevented due to the effects of manuka honey on autolysin activity, which normally controles the septum rupture to produce the two daughter cells [24].

In addition, it was corroborated that manuka honey induced a dose-dependent extension of the lag phase of cell growth of *B. subtilis*, *E. coli,* and *S. aureus* when they were treated with manuka honey. This growth behavior was also observed when these bacterial cultures were exposed to MGO, and was not detected after the treatment of bacteria with other honey varieties (clover or kanuka honey); so, the extended duration of lag phase was supposed to be due to MGO [66]. In contrast, *P. aeruginosa* showed a markedly different pattern of growth inhibition to the other three bacteria; very little or no lag-phase extension was observed after honey treatment, except for the treatment with clover honey [66].

Finally, honey also affects bacterial DNA. This effect, observed on *B. subtillis* and *E. coli*, was related to the interactions of H_2_O_2_ with polyphenols as active intermediates that are necessary to confer oxidative action of H_2_O_2_ through hydroxyl radical production [37]. This mechanism was supported by transmission electron microscopy observations, which revealed “ghost” *E. coli* cells lacking DNA, after citrus honey treatment [68]. Moreover, exponentially growing *P. aeruginosa* cells presented condensed chromosomes after treatment with manuka honey, which suggest that this variety of honey inhibits DNA replication in these cells by non-oxidative stress mechanisms [25,66].

### 3.4. Disruption of Bacterial Metabolism

Despite the fact that very few studies have been carried out to evaluate the effect of honey on the metabolic activity of bacteria, the concordance of the results obtained indicates that honey plays a key role in the metabolic disruption of both Gram-positive and Gram-negative bacteria. Genomics and proteomics demonstrated that manuka honey is able to reduce the expression of genes and proteins involved in the energy metabolism of MRSA [73]. These findings were corroborated by other flow cytometry assays to evaluate the repercussion upon metabolic bacterial physiology of reference strains of *S. aureus* and *E. coli* induced by manuka honey, and also by other three honey varieties (avocado, chestnut and polyfloral) [47,64]. However, differences in bacterial response, depending on the honey type and the concentration tested, were observed, probably due chemical composition variances among samples. Metabolic disruption induced by manuka honey seemed to be an irreversible effect, whereas in the case of the other honey varieties, bacteria appear to reprogram their metabolism in response to the environmental stress, being able to progressively recover metabolic activity, at least partially, at the end of honey exposure [47].

Moreover, other underlying mechanisms could also affect bacterial metabolism. Studies on *E. coli* [74,75], as well as on *P. aeruginosa* [75,76] and *S. aureus* [75], confirmed the effect of manuka honey as an iron-chelator, generating a limiting environment of this element, which is essential for bacterial metabolism and survival. Furthermore, membrane potential is also a fundamental process in energy generation for bacteria. It has been described that honey induces membrane depolarization [25,47,64], which may impede the ability of bacteria to generate the energy required for several processes [25].

### 3.5. Effect on Efflux Pumps Activity

Multidrug efflux pumps are cell membrane glycoproteins that can eject different classes of compounds across bacterial membranes; for this reason, they are one of the most important mechanisms of drug resistance, since antibiotics are not capable of reaching the concentration at which they are active within the bacteria [77,78].

Some natural products, including plant extracts, essential oils or isolated compounds, are capable of inhibiting or modifying the efflux pumps’ activity [78]. In this context, manuka honey has been proven to induce alterations in the expression of genes belonging to the e*vg*AS regulon, related to bacterial adaptive responses to acid, osmotic and drug resistance [74]. The results obtained in this first study were partly corroborated by later research; another study using different honey types (clover, citrus and marjoram) demonstrated that depending on the honey variety, the gene expression profile was different. *EvgA* expression was upregulated after the treatment with clover honey, but it was downregulated after citrus or marjoram honey-exposure. [68]. The dissimilarities in the expression patterns may reflect compositional differences among honey varieties and/or differences in their action mechanisms, since the major antimicrobial activity of manuka honey is not related to H_2_O_2_, while the other honey varieties were shown to be mainly peroxide-dependent [68].

A more recent study, using flow cytometry, proved that manuka honey was able to disrup efflux pump activity of an AG100 progeny strain of *E. coli,* which over-expresses several efflux pumps when exposed to high concentrations of tetracycline. Manuka honey blocked efflux pump activity, following a dose-dependent tendency [64]. This finding seems to differ from those previously described. These discrepancies may be justified by a lack of correlation between gene expression and bacterial physiological response or by other mechanisms not directly related to the genes involved in bacterial defence and adaptative reponse. It was suggested that this effect could be associated with the metabolic disruption, since antibiotic efflux is an energy-dependent mechanism [64]. More recently, Bouza et al. hypothesized that manuka honey could increase the drug uptake due to the membrane potential collapse, and the increase in membrane permeabilization, which together would enhance the potency of antibiotics [25]. These mechanisms justify why manuka honey is able to restore the tetracycline antibiotic activity against bacterial strains that would otherwise be resistant [25,79].

### 3.6. Bacterial Quorum Sensing Alterations

Quorum sensing is the cell to cell communication system used to coordinate and regulate the behavior of cell populations. Many bacterial physiological functions, such as luminescence, virulence, motility, sporulation or biofilm formation, are regulated by quorum sensing systems [20]. For this reason, the interruption of bacterial quorum sensing is recognized to alleviate virulence, and is considered to be a potential new alternative to treat infections caused by pathogenic bacteria [20].

Only a few studies on the effect of honey on inhibition of quorum sensing have been published. Nevertheless, the results demonstrate that some genes involved in this bacterial interconnection mechanism, as well as other virulence genes, are downregulated following exposure to honey [21,25,68,73,80]. Furthermore, honey was able to enhance the anti-quorum sensing activity of other substances, such as curcumin, when they were used in combination [81].

Additionally, the secretion of *N*-acyl-l-homoserine lactones and other quorum sensing regulating or signalling substances (pyocyanin, pyochelin and pyoverdine) is significantly reduced in response to honey exposure [20,81]. It was suggested that this inhibitory activity seems to be related to the aqueous phase of the honey instead of either the total or individual phenolic content [20] (Figure 1). In addition, pyocyanin, pyochelin and pyoverdine act as iron-chelating molecules or siderophores, which are central to bacterial proliferation in the host environment, and are inherently linked to pathogen virulence [13,76]. Honey-treatment mediates a marked reduction in siderophore production, which is accompanied by the reduction in the expression of genes involved in *quorum sensing.* This fact demonstrates the global impact that gene downregulation exerts on the expression of virulence factors [13].

### 3.7. Biofilm Inhibition

Biofilms constitute an extraordinary microbial drug-resistance mechanism, which is composed of an assemblage of microbial cells that are irreversibly associated with a surface and enclosed in a matrix of self-produced polysaccharide material. Included in biofilms, bacteria are several times more resistant to antibiotics than planktonic cells, which makes them extremely difficult to destroy [82,83].

The effects of different varieties of honey samples on bacterial biofilms have been assessed in various studies, and the results indicate that honey is effective in disturbing the biofilm strengthening in drug-sensible and drug-resistant bacteria.

First of all, honey is able to prevent the formation of biofilms, from both Gram-negative and Gram-positive bacterial species, most likely via a non-specific mechanism such as the inhibition of bacterial growth, and the reduction in the biofilm biomass [22,36,82,84,85,86,87], which are consequences of the osmotic stress, coupled with the low pH and the presence of H_2_O_2_ of honey (Figure 1). This effect is dependent on concentration—when honey concentration is not enough, the biofilm formation could be enhanced [36,87].

In addition, when the biofilm is already established, honey significantly reduces its metabolic activity, probably due to the disruption of the metabolic activity of individual bacterial cells—this mechanism is also suggested to explain the inhibition of biofilm development when it is forming [36,88]. In this phase, honey also decreases the number of viable cells [36,82,84,85,87].

Finally, gene expression analysis also confirmed the effect of honey on the expression of different genes related to the formation and the development of biofilms [68]. Honey might prevent the colonization of host tissues due to the downregulation of genes encoding binding proteins [73,85,88], and the inhibition of the expression of genes encoding a glucosamine polymer [19].

### 3.8. Effects on Bacterial Stress Response

Several studies have demonstrated that honey exposure also affects the expresion of different genes involved in bacterial stress response. Manuka honey exposure generates the downregulation of the universal stress protein UspA in MRSA, which reduces the ability of bacteria to survive under cellular and metabolic stress conditions [89].

In addition, other studies have demonstrated similar effects in genes related to cell stress survaival mechanisms, namely *rpoS* and *H-NS*, after the exposure of *E. coli* cells to citrus, clover, and marjoram honey samples [68]. These results were in contrast to those previously described by Blair et al., who showed marked upregulation in the same genes of *E. coli* following manuka honey-treatment [74]. A more recent study demonstrated that manuka honey induced strong upregulation in a wide range of genes involved in the emergencyand the oxidative stress response of *P. auregonisa* cells [25].

Taking into account all these results, it is feasible to think that the variances in expression pattern may be justified by the differences in the antimicrobial mechanisms of the honey varieties tested, and the variable effects they can induce on certain genes.

## 4. Honey against Antibiotic-Resistant Bacteria

It is well demonstrated that honey inhibits a broad range of bacteria, and is equally as effective against antibiotic-susceptible bacteria as it is against those that are resistant [13,30,90,91,92]. Nevertheless, since all the results obtained from in vitro studies always cannot be reproduced under in vivo conditions, very few studies of this type have been undertaken (Table 1).

Paradoxically, one of the first assays to test the efficacy of honey against antibiotic-resistant microorganisms was carried out in vivo, to heal a hydroxyurea-induced leg ulcer colonized by MRSA, using manuka honey. This study recognized that honey possesses antibacterial properties upon resistant bacteria, and can also promote effective wound healing [93]. Following this study, a significant amount of work using manuka honey confirmed its effects against MRSA in vitro [24,94,95,96,97,98,99,100] and in vivo [101], vancomycin-resistant *S. aureus* [91], and vancomycin-resistant enterococci (VRE) [100]. MRSA and VRE are considered the foremost pathogens that often infect chronic wounds in the earlier stages [96]. In Gram-negative bacteria, the effects of manuka honey has also been observed against extended-spectrum beta-lactamase (EBSL)-producing *E. coli* [94,102], ESBL-producing *Klebsiella pneumoniae* [94,103,104], and carbapenemase-producing *Klebsiella pneumoniae* [94], as well as against multidrug-resistant *P. aeruginosa* [94]. Manuka honey has also demonstrated antimicrobial activity against *Ureaplasma parvum* and *Ureaplasma urealyticum,* pathogens without a cell wall with high levels of intrinsic and acquired antibiotic resistance [105]. Moreover, preceding in vivo studies confirmed the efficacy of Medihoney (the medical grade manuka honey) in the prevention of catheterisation-associated infections [106].

Manuka honey has undoubtedly been shown to be effective against a broad spectrum of antibiotic-resistant bacteria, and it is the variety most used in antimicrobial activity assays. For these reasons it is commonly used as “control honey”. However, studies with other varieties confirm that this kind of honey is not the only one with potentialities in the fight against resistant bacteria (Table 1).

Another medical-grade honey, Revamil, showed antibacterial activity against various antibiotic-resistant wound pathogens [60,61,107]. The data indicated in these studies that the antibiotic sensitive counterparts for the tested microorganisms were no more or less susceptible to honey treatment. However, bactericidal effects required prolonged honey-exposure of the target microorganism [61]. In addition, in vivo studies demonstrated that this type of honey was able to reduce the microbial colonization of skin of healthy volunteers 100-fold [107]. More recently, L-Mesitran products, formulated with medical-grade honey, demonstrated in vitro their activity against MRSA [99], and in vivo their promising applicability to treat chronic diabetic ulcers, preventing amputations derived from antibiotic-resistant infections [108].

Apart from manuka honey and medical-grade-honey-based formulations, other works proved that “common” honey samples play an important role as antibacterial agents too. *E. coli*, *S. aureus*, and different species of genus *Bacillus* and *P. aeruginosa* resistant to several antibiotics, were susceptible, in vitro, to honey from aromatic and medicinal plants from Morocco [109].

After this early work, the scientific community realized the potential of different honey types in the treatment of antibiotic-resistant bacteria, and numerous studies screened different honey varieties from all over the world. Ulmo tree honey induced greater inhibitions zone and showed lower minimal inhibitory and minimal lethal concentrations than manuka honey against reference and clinical isolates of MRSA. The activity of this honey type was attributed to its strong H_2_O_2_ antimicrobial effect [110]. To provide a better insight into the involvement of H_2_O_2_ on bacterial growth, Brudzynski and Lanniganwe tested different varieties of Canadian honey (sweet clover, blueberry, and buckwheat, previously selected for their higher bacteriostatic activity), against MRSA and vancomycin-resistant *Enterococcus faecium* [111]. All bacterial strains were sensitive to honey bacteriostatic action. The study also confirmed that the generation of hydroxyl radical from the H_2_O_2_ which is present in honey caused growth inhibition of the both clinical isolates [111].

The effectiveness of black seed, beri and shain honey in treating twenty-five wound isolates of MRSA and other wound pathogens was compared to manuka honey efficacy [112]. Manuka honey showed better antibacterial activity against all tested clinical isolates as compared to the other varieties. However, the three varieties revealed good in vitro performance (MIC values did not exceed 11% (*w/v*)), and black seed honey exhibited a comparable antibacterial activity to manuka honey [112].

Different Scottish honey samples (blossom, heather, Highland, and Portobello Orchard) exhibited antimicrobial activity upon clinical isolates of penicillin-resistant *Acinetobacter calcoaceticus*, *S. aureus*, *P. aeruginosa* and *E. coli* [38]. These honey varieties were not as effective as manuka honey in inhibiting bacterial growth. However, Highland honey and Portobello honey showed good results. Antimicrobial activity appeared to be influenced, but not completely explained, by phenolic compound content. Characteristic polyphenol components in the different honey samples, as well as novel glycoside derivatives of fatty diacids, which may contribute to their antimicrobial activities, were found [38].

**Table 1:** Different honey varieties and honey-based products against antibiotic-resistant bacteria, type of study accomplished (in vitro or in vivo) and reference number of the studies from which the information was extracted.

Honey Type.	Microorganism	Type of Study	Reference
Manuka	MRSA	in vivo hydroxyurea-induced leg ulcer leg ulcer	[93] [101]
		in vitro	[24,69,94,95,96,97,98,99,100,113]
	VRSA	in vitro	[91]
	VRE	in vitro	[100]
	ESBL-*E. coli*	in vitro	[94,102]
	ESBL-*K. pneumoniae*	in vitro	[94,103,104]
	CARB-*K. pneumoniae*	in vitro	[94]
	MDR-*P. aeruginosa*		
	*Ureaplasma parvum* *U. urealyticum*	in vitro	[105]
Medihoney	Bacteria related to catheterization infections	in vivo catheterization infections	[106]
Revamil	MRSA MRSE ESBL-*E. coli* ESBL-*P. aeruginosa* ESBL-*E. cloacae* ESBL-*K. oxytoca* VREF	in vitro	[60,61,107]
	Colonizing skin bacteria	in vivo skin colonization	[107]
L-Mesitran	MRSA	in vitro	[99]
	MDR-*P. aeruginosa* MDR-streptococci AR-polymicrobial infection PR-polymicrobial infection MDR-*E. coli*	in vivo chronic diabetic ulcers	[108]
Aromatic-and-medicinal-plants honey	MDR-*E. coli*	in vitro	[109]
	MDR-*S. aureus*		
	MDR-*Bacillus* spp.		
	MDR-*P. aeruginosa*		
Ulmo tree	MRSA	in vitro	[110]
Sweet clover Blueberry Buckwheat	MRSA VREF	in vitro	[111]
Black seed Beri Shain	MRSA	in vitro	[112]
Blossom Heather Highland Portobello	PR-*A. calcoaceticus* PR-*S. aureus* PR-*P. aeruginosa* PR-*E. coli*	in vitro	[38]
Sage Chestnut Locust tree Lime tree Indigo bush Rapeseed Maple Mint Spring and Autumn pasture Maple Fir honeydew	MRSA MDR-*P. aeruginosa* ESBL-*E. coli* MDR-*A. baumannii*	in vitro	[114]
Agastache Tea-tree Jelly bush Jarrah	MRSA	in vitro	[69]
Avocado Chestnut Eucalyptus Rosemary Thyme Polyfloral	MRSA MDR-*S. pyogenes* MDR-*E. coli* MDR-*P. aeruginosa*	in vitro	[30]
Germania	MRSA	in vitro	[113]

MRSA: methicillin-resistant *S. aureus*; VRSA: vancomycin-resistant *S. aureus*; VRE: vancomycin-resistant enterococci; ESBL: extended-spectrum beta-lactamase-producing; CARB: carbapenemase-producing; MDR: multi-drug resistant; MRSE: methicillin-resistant *S. epidermidis*; VREF: vancomycin-resistant *Enterococcus faecium*; AR: ampicillin-resistant; PR: penicillin-resistant.

In the case of diverse Croatian honey samples (sage, chestnut, locust tree, lime tree, indigo bush, rapeseed, maple, mint, Spring and Autumn pasture, and maple and fir honeydew honey samples), variable effects upon bacterial strains of MRSA, multidrug-resistant *P. aeruginosa,* ESBL-producing *E. coli* and multidrug-resistant *Acinetobacter baumannii* were observed [114]. Fir honeydew and mint honey were the honey varieties which showed significantly higher antibacterial activity against all bacteria. On the other hand, it was assumed that locust tree, rapeseed and spring pasture honey samples did not have the necessary ingredients or synergy among active components to affect bacteria [114].

Afterwards, antimicrobial activity of agastache honey was compared with those of manuka, tea-tree, jelly bush, super manuka (MGO-400), and jarrah honey [69]. All samples showed some antibacterial activity upon reference and clinical isolates of MRSA, as well as upon other wound pathogens. However, inhibitory and bactericidal effects varied, depending on the bacterial species, strain, and the type of honey; the effective concentrations ranged from 3.12% to 25% (*w/v*). Hydrogen peroxide contributed to the antimicrobial activity of agastache, super manuka, and jarrah honey, but not in manuka and jelly bush honey, while the tea tree honey activity was largely H_2_O_2_-based [69].

Spanish honey samples of several botanical origins (avocado, chestnut, eucalyptus, rosemary, thyme, polyfloral) also demonstrated in vitro efficacy against MRSA and other pathogenic bacteria (*Streptococcus pyogenes, E. coli,* and *P. aeruginosa*) with varying degrees of antibiotic-resistance. However, effective concentrations were rather variable, ranging between 0.05 and 0.40 g/mL, depending on the honey variety and the microorganism tested [30].

One of the last studies to evaluate the antibacterial potential of honey showed that not all honey varieties have a good performance. In vitro diluted Germania honey was relatively ineffective against MRSA, and was significantly less effective than manuka honey even when undiluted [113]. This study highlights the importance of testing and selecting an adequate variety of honey, especially when it is going to be applied for clinical purposes.

## 5. What about Honey-Resistance Acquisition?

One of the biggest conundrums in using an antibiotic is how long it will be effective in treating the target microorganism. Usually, antibiotics or chemotherapeutic substances aim at a particular process, structure or physiological mechanism, and in this way provide a specific evolutionary pressure to which bacteria are easily able to adapt [13]. Honey is a natural substance with a complex composition, which turns out to be one of the main advantages for its use as an antimicrobial agent. The presence of hundreds of compounds, which might act additively or synergistically in multiple bacterial targets, makes it difficult for bacteria to adapt to them, and therefore, make the development of resistance more complicated [13,87].

Some studies have been carried out using manuka honey to evaluate the possibility that its extensive use might provide a selective pressure for the emergence of honey-resistant bacteria. Blair et al. were the first in demonstrating that *S. aureus* and *P. aeruginosa* did not acquire resistance to honey when they were continuously exposed to sub-lethal concentrations of manuka honey. This study refuted the theory that bacteria could rapidly gain resistance to MGO, as the main antimicrobial substance present in manuka honey [74]

Afterwards, Cooper et al. accomplished in vitro assays with reference to clinical isolates of different bacteria by repeated cultivation in a sub-lethal concentration of honey for ten consecutive days, or by incubation in stepwise increasing concentrations over twenty-eight days. The results indicate that it was not possible to generate honey-resistant mutants in a short-term stepwise resistance training period. The same results were observed when bacteria were exposed to increasing concentrations of honey in a long-term assay. However, the honey MICs increased for *P. aeruginosa*, MRSA, *Staphylococcus epidermidis* and *E. coli* by factors of 1.4, 1.6, 1.7 and 2.1, respectively— even so, these changes were not persistent [115].

More recently, Mokhtar et al. evidenced that long-term exposure of different strains of *S. aureus*, *S. pyogenes*, *P. aeruginosa, E. coli*, MRSA and *S. epidermidis* to manuka honey was not able to generate isolates with a reduced susceptibility to honey following a similar time frame in which resistance to macrolides was generated [97]. Moreover, it was suggested that the iron chelation capacity of manuka honey affects multiple diverse physiological processes in bacteria and would contribute to the lack of bacterial resistance to honey [75].

Despite all these encouraging findings, it is necessary to take into account the results reported by Camplin and Maddocks, which demonstrate that *P. aeruginosa* isolates that recovered from honey-treated in vitro biofilms can develop sustained, increased resistance, which might be due to the appearance of small colony variants within the microbial population [116].

In addition, it has been observed that honey acts synergistically with several antibiotics, reducing the doses required to inhibit bacterial growth or reverting the antibiotic-resistance previously acquired. The synergism or additive effects between manuka honey and tetracycline, imipenem, mupirocin and gentamycin has been described for *P. aeruginosa* [79,117], as well as between manuka honey and oxacillin, rifampicin, linezolid, tetracycline, imipenem, mupirocin and vancomycin for susceptible *S. aureus* and MRSA [63,79,95,98,117,118], which demonstrates that manuka honey interacts positively with different antibiotic classes to inhibit bacterial growth. In addition, in combination with oxacillin, manuka honey was able to restore the susceptibility of MRSA to the antibiotic [95]. The mechanisms by which synergism occurs have not been described in detail. It has been suggested that this could be, at least partly, due to an increase in hydroxyl radical production due to the presence of honey. However, other factors must be involved because peroxide activity in manuka honey is not essential for the complete inhibition of bacterial growth [63]. Moreover, the presence of MGO increased the intracellular accumulation of the antibiotic, enhancing its activity against bacteria [118].

Further, manuka honey-antibiotic synergism also affects biofilm formation, especially with rifampicin [63,119]. Since manuka honey alters the levels of protein-synthesis components, including ribosomal proteins, this effect is probably due to a ‘like plus like’ effect on the protein synthesis pathway [63,74,90].

## 6. Limitations of the Use of Honey in Therapy

The extensive scientific evidence proves that honey may offer distinct advantages over the chemotherapeutic substances currently used. However, despite being a potential antimicrobial agent, its use in medicine remains underestimated due to a series of limitations, mainly related to compositional and application aspects.

Honey is a natural substance with a rather variable composition, which depends primarily on its botanical origin [30]. Compositional variances condition its bioactive potential and hamper its further application for clinical purposes [120,121]. This fact highlights the importance of selecting an appropriate variety of honey, which means that a previous screening is necessary in order not only to quantify but also to determine profiles of bioactive substances [19,30]. In addition, it is necessary to consider that, although minor, there is a possibility that honey might induce negative effects, either by ingestion [122,123] or by topical use, mainly related to the presence of *Clostridium botulinum* spores, which have occasionally been found in honey [19,124,125]; however, to date, no cases of wound infection due to *C. botulinum* spores related to the use of non-irradiated honey were reported. Moreover, honey for medical uses must be free of any form of contamination, such as herbicides, pesticides, heavy metals, and spores—to meet all these criteria, honey must be collected in organic regions, as well as following strict quality, processing, and storage standards and regulations, in order to ensure its safety [108].

On the other hand, the absence of standardization of antibacterial activity, and the incomplete knowledge of the active components, and how they interact and act against bacteria, are the major limitations for the application of honey in medicine [61,126]. So, more studies attempting to detail the antibacterial effects of the different components present in honey, as well as to elucidate the molecular mechanisms underpinning the observed effects, are necessary. In addition, robust, large-scale clinical trials are imperative to confirm that honey’s efficacy is similar in vitro to that in in vivo assays. In this regard, the feasible application of honey in medicine should be considered. As an antibacterial agent, honey is not a systemic therapy—to produce an effect, it must be in contact with the target microorganism, which restricts it use mainly to treat wounds infections and as a topical preventive at sites where microorganisms could cause an infection [13,14,107,126].

Some of the limitations described are overcome by medical-grade honey varieties, which have been approved as medical products for wound care [61]. Medical grade honey is sterilized, produced under rigorous standards of hygiene, without contaminating pesticides or pollutants in its composition, and standardize under different defined criteria [96].

## 7. Conclusions

The emergence and rapid spread of antibiotic-resistant bacteria is one of the most pressing concerns not only for human health, but also for human-connected animals, farms, food, water, and natural ecosystems worldwide. The limitations posed by the development and commercialization of new-generation antibiotics have led to a relative absence of effective options on the market and have forced the search for new alternatives. Numerous scientific studies are being carried out to evaluate the antimicrobial potential of different substances, formulas or active ingredients traditionally used before the advent of antibiotics, and honey is one of them.

Honey has been used since ancient times for its nutritional and medicinal properties, especially for its antimicrobial activity. Several studies have scientifically demonstrated its efficacy against pathogenic bacteria, both sensible-and-resistant to antibiotics. In its composition, honey presents hundreds of compounds which act upon several target sites, additively or synergistically, since when used in isolation, at the concentrations found in honey, many of them do not produce effects upon bacteria. This characteristic could be behind the difficulty of bacteria to acquire honey-resistance. In addition, the combined used of honey and antibiotics reduces the concentrations of drugs necessary to achieve efficacy, thus limiting the likelihood of developing resistance, and reverting the susceptibility of some resistant bacteria to antibiotics.

All these assets suggest that honey might find a place in clinical practice as part of combination antimicrobial therapies with systemically administered antibiotics to treat multidrug-resistant bacteria, especially in topical applications. However, the honey used for medical purposes must be guaranteed, in its antibacterial efficacy and its safety for patients.
